# The effects of an individualized smartphone-based exercise program on self-defined motor tasks in Parkinson’s disease: a long-term feasibility study

**DOI:** 10.1186/s41687-023-00631-6

**Published:** 2023-10-30

**Authors:** Lisa Lützow, Isabelle Teckenburg, Veronika Koch, Franz Marxreiter, Jelena Jukic, Sabine Stallforth, Martin Regensburger, Jürgen Winkler, Jochen Klucken, Heiko Gaßner

**Affiliations:** 1 Department of Molecular Neurology, University Hospital Erlangen, Friedrich-Alexander University Erlangen-Nürnberg (FAU), Schwabachanlage 6, 91054 Erlangen, Germany; 2 Medical Valley – Digital Health Application Center GmbH, Bamberg, Germany; 3 Fraunhofer Institute for Integrated Circuits IIS, Erlangen, Germany; 4 Center for Movement Disorders, Passauer Wolf, Bad Gögging, Neustadt an der Donau, Germany; 5 Present Address: Luxembourg Centre for Systems Biomedicine, University of Luxembourg, Esch-sur-Alzette, Luxembourg; 6 Present Address: Luxembourg Institute of Health, Strassen, Luxembourg; 7 Present Address: Centre Hospitalier de Luxembourg, Luxembourg, Luxembourg

## Abstract

**Background:**

Exercise therapy is considered effective for the treatment of motor impairment in patients with Parkinson’s disease (PD). During the COVID-19 pandemic, training sessions were cancelled and the implementation of telerehabilitation concepts became a promising solution. The aim of this controlled interventional feasibility study was to evaluate the long-term acceptance and to explore initial effectiveness of a digital, home-based, high-frequency exercise program for PD patients. Training effects were assessed using patient-reported outcome measures combined with sensor-based and clinical scores.

**Methods:**

16 PD patients (smartphone group, SG) completed a home-based, individualized training program over 6–8 months using a smartphone app, remotely supervised by a therapist, and tailored to the patient’s motor impairments and capacity. A control group (CG, n = 16) received medical treatment without participating in digital exercise training. The usability of the app was validated using System Usability Scale (SUS) and User Version of the Mobile Application Rating Scale (uMARS). Outcome measures included among others Unified Parkinson Disease Rating Scale, part III (UPDRS-III), sensor-based gait parameters derived from standardized gait tests, Parkinson’s Disease Questionnaire (PDQ-39), and patient-defined motor activities of daily life (M-ADL).

**Results:**

Exercise frequency of 74.5% demonstrated high adherence in this cohort. The application obtained 84% in SUS and more than 3.5/5 points in each subcategory of uMARS, indicating excellent usability. The individually assessed additional benefit showed at least 6 out of 10 points (Mean = 8.2 ± 1.3). From a clinical perspective, patient-defined M-ADL improved for 10 out of 16 patients by 15.5% after the training period. The results of the UPDRS-III remained stable in the SG while worsening in the CG by 3.1 points (24%). The PDQ-39 score worsened over 6–8 months by 83% (SG) and 59% (CG) but the subsection mobility showed a smaller decline in the SG (3%) compared to the CG (77%) without reaching significance level for all outcomes. Sensor-based gait parameters remained constant in both groups.

**Conclusions:**

Long-term training over 6–8 months with the app is considered feasible and acceptable, representing a cost-effective, individualized approach to complement dopaminergic treatment. This study indicates that personalized, digital, high-frequency training leads to benefits in motor sections of ADL and Quality of Life.

**Supplementary Information:**

The online version contains supplementary material available at 10.1186/s41687-023-00631-6.

## Introduction

Parkinson’s disease (PD) is the second most common neurogenerative disease with rapidly increasing prevalence [1–3]. It is characterized by a symptom complex including tremors, rigor (stiffness of joints) and akinesia due to the loss of dopaminergic neurons in the substantia nigra. The guideline-based treatment consists of dopaminergic drugs complemented by physical therapy and exercise therapy [4, 5].

### Complementary high-frequency exercise therapy in PD

Although medical treatment has led to improved symptom control and higher life expectancies for PD patients, curative treatment remains unavailable. Living with increasing symptoms is viable, paired however with a substantial decrease in quality of life [6]. The role of rehabilitative management and complimentary therapy continues to grow, complimented by several studies proving the effectiveness of exercise therapy in PD patients [4, 7–11]. In some home-based concept studies (e.g. treadmill training [12], virtual reality balance training [13], and aerobic exercises [14]), it has been found that when included alongside pharmacological treatment, these therapies can improve PD-related motor impairments, such as gait, balance, strength and physical capacity [15, 16]. Since high-frequency training (4 times per week) is recommended, outpatient options need to be explored to facilitate sustainability and limit required organizational effort [11, 12, 17–19]. However, the choice of exercises and optimal intensity depend on the impairments of each patient and should be tailored by therapists with expertise in PD [14, 19, 20].

### Impact of the COVID-19 lockdown on PD patients

The COVID-19 pandemic presented an unprecedented challenge to the global health system leading to the cancelation of elective surgeries and therapy sessions. PD patients, belonging to a particularly vulnerable groups with a higher risk for severe COVID-19 outcomes, limited their social contacts, stayed at home, and cancelled their physiotherapy sessions [21, 22]. Most reported increased motor and non-motor symptoms as well as a reduction of their Quality of Life (QoL) emerging from physical inactivity and social isolation [23–25].

### The implementation of telerehabilitation

Consequently, the COVID-19 pandemic caused a massive acceleration in the use and development of telehealth solutions enabling patients to overcome organizational or location-based barriers.

In several studies, home-based tele-exercise training was found to be comparably effective and accepted as conventional exercise training [26, 27]. PD patients exhibit the motivation and ability to engage in new digital and remote therapies [28–30]. The implementation of home-based exercise training via smartphone apps or video conferences may also include remote supervision and modification by trained professionals and therapists. Maintaining the balance between individual flexibility and professional supervision, smartphone applications resemble a cost-efficient solution that could be implemented in routine care and offered to all patients.

A previous interventional pilot study showed that an individualized, app-guided, and high-frequency exercise program is feasible in PD patients over a time of 4 weeks [31]. The improvement of patient-defined motor tasks of 40% on average indicated that remotely-supported, digital exercise training has the potential to reduce motor symptoms. Though the benefits and potentials of telerehabilitation are clear, further investigation of long-term acceptance, usability and feasibility is still needed to establish smartphone-based, personalized training concepts in routine care.

### Patient-reported outcome measures to rate therapy effectiveness

Patient-reported outcome measures (PROMs) represent the patient’s individual perception of their health outcomes such as functional status, QoL or disease symptoms [32]. PROMs are already used to track symptoms or monitor outcomes internationally but have yet to be implemented in routine care [32–34].

PROMs have gained relevance highlighting the potential of shared-decision making and value-based health care at the individual patient level [35, 36]. Despite the abundant acknowledgment of outcome-orientated customized therapy, there is still a lack of applications offering home-based exercise programs tailored to the individual, prioritising the unique impairments of each patient.

Our intention was to bridge the gap between the symptom progression described by the participants and the objectifiable clinical outcomes measured by standardized tests, while similarly constructed studies solely focused on the latter [37]. Combining patient-centered, high-frequency exercise training and the use of PROMs, we aimed to improve daily motor activities directly affecting QoL. After the 6-to 8-months lasting feasibility study we evaluated the acceptance and effectiveness of an individualized, remotely-supervised exercise program that would give many PD patients access to high-frequency training.

## Methods

This study is a prospective controlled interventional trial that focuses not just on clinical measures of successful outcome but also the improvement of patient-defined most restrictive motor symptoms.

### Study cohort

In this study, 17 patients diagnosed with sporadic PD according to the Movement Disorder Society Clinical Diagnostic Criteria in Hoehn&Yahr (H&Y) stages 1 to 4 were recruited from the Movement Disorder Outpatient Unit at the Department of Molecular Neurology (University Hospital Erlangen, Germany) [38, 39]. Patients with motor fluctuations or substantial cognitive impairments inhibiting them from following instructions or restricting their ability to properly use a smartphone were excluded. All patients proceeded with their medical treatment and maintained normal daily activities throughout the study. More detailed information is presented in the patient characteristics (Table 1).

**Table 1:** Characteristics of PD patients at baseline (T0)

	SG (n = 17)	CG (n = 16)	*p*
	mean ± SD	mean ± SD	
Age, years	64.8 ± 7.8	64.8 ± 8.3	0.987
Gender, male/female	11/6	10/6	
Height, cm	176.4 ± 8.8	171.5 ± 11.7	0.182
Weight, kg	85.3 ± 14.2	82.9 ± 13.5	0.634
BMI, kg/m^2^	27.4 ± 4.5	27.9 ± 3.2	0.713
Disease duration, years	8 ± 6	8.1 ± 5.0	0.974
LEDD, mg/d	765.3 ± 403.3	823.5 ± 356.4	0.664
Hoehn & Yahr	2.0 ± 0.6	2.1 ± 0.4	0.725
UPDRS-III	15.3 ± 9.2	12.6 ± 6.6	0.725
PDQ-39	12.9 ± 14.1	15.4 ± 9.6	0.581
MoCA	25.7 ± 2.9	26.3 ± 2.3	0.555

*SD* Standard deviation, *LEDD* Levodopa equivalent daily dose, *UPDRS-III* Motor score of the Unified Parkinson’s disease rating scale, *MoCA* Montreal Cognitive Assessment

11 out of 17 patients already participated in the pilot study in 2020 [31]. Due to a non-related SAE, the dataset of one patient could not be completed. Therefore, the statistical analysis includes data from 16 patients in the smartphone group (SG).

This study was approved by the local ethics committee (reference number: 72_20 B, Medical Faculty, FAU Erlangen-Nürnberg, Germany). All participants provided written informed consent prior the first study assessments.

### Control group

16 patients with sporadic PD selected from the database of the Movement Disorder Unit at the Department of Molecular Neurology (University Hospital Erlangen, Germany) were age and gender-matched with the study cohort. All data and tests were collected from their regular doctor visits in 2019 and 2020. Patients received best medical treatment, including dopaminergic medication according to their stage of disease, and reported no substantial change of their exercise behaviour during the investigated period. Since they did not participate in any smartphone-based exercise training, symptom progress in the CG represents the natural course of PD in a modern health system. Due to the reduction of clinical visits during the Covid19-pandemic, the examined time period of the control group (CG) was significantly longer than the duration of the smartphone-based intervention.

### Assessments

All assessments were conducted during the baseline examination (T0) and were repeated after a 10-week training period (T1, intermediate examination), and a 26-week training period (T2, post examination).

#### Questionnaires and scales

Questionnaires about the patient’s fear of falling (FES-I), frequency of falls (FFQ), and Parkinson-specific quality of life (PDQ-39) were sent to the patients before the pre-examination and collected during the study [40–43]. Motor impairment was rated by a certified movement disorder specialist using the motor subscale of the Unified Parkinson’s Disease Rating Scale (UPDRS-III) and cognitive impairments were evaluated using the Montreal Cognitive Assessment (MoCA) [44, 45].

#### Patient-reported outcome measures

PD patients of the SG were also asked to name all daily life activities (M-ADL) that are affected by their motor symptoms and to assess the impairment on a scale of 0 (no restrictions) to 10 (maximum impairment) during each examination. A discrete interval scale running in steps of 0.5 was used and the results were presented in radar plots. The area outlined by the curves was calculated and compared using the formula

$$\mathop \sum \limits_{{\text{i}}} {\text{F}}_{{\text{i}}} = \mathop \sum \limits_{{\text{i}}} \frac{{{\text{x}}_{{\text{i}}} \cdot {\text{x}}_{{{\text{i}} + 1}} \cdot {\text{sin}}\left( {\upgamma } \right)}}{2}$$

In the formula ${\text{x }}$ represent the points on the scale reported by the patients. The angle γ is received by dividing 2π by ${\text{ i}}$ (the number of points measured). Since one patient only named two activities, the formula could not be used in this case. The relative and absolute amount of change was calculated by the sums of the stated values before and after the training period.

#### Classification in subgroups

The SG was divided into two subgroups according to the change of the self-recorded motor activities of daily living (M-ADL) from T0 to T2. The group of Responder (R) included 10 patients who stated less motor impairment after the training period and therefore improved their M-ADL scores. The 6 patients that showed an increase of impairment (higher amount of M-ADL) were defined as the group of Non-Responder (NR) since they did not respond to the training program according to their M-ADL scores. A detailed characterization of both subgroups by their baseline results can be found in the Additional file 1: Table 1.

#### Sensor-based assessments

Sensor-based gait analysis included a standardized 4 × 10 m walking test, the Time Up and Go Test (TUG) and a 2 min walking test (2MWT). Detailed information about the sensors is presented in the supplements. More comprehensive information on sensor-assisted gait analysis and the validation of the selected gait tasks can be obtained from previous work [46–50].

#### Evaluation of usability

Feasibility and usability of the app was assessed using the System Usability Scale (SUS) and the User Version of the Mobile Application Rating Scale (uMARS) at the end of the study [51–53]. The SUS is a commonly used method for measuring the usability of applications and other technological products. The uMARS is a 5-point rating scale *(1-inadequate, 2-poor, 3-acceptable, 4-good, 5-excellent)* measuring the usability of mobile health apps.

### Smartphone-based exercise program

The smartphone application “PatientConcept” (NeuroSys GmbH, https://patientconcept.app/) provides exercise training tailored to the personal needs and capacity of PD patients. It includes 50 short training videos focused on balance, flexibility, strength, gait, balance, posture, coordination and rhythm, large amplitude movements, and fine motor skills. All patients received a tailored program including 3 to 9 video sequences that were chosen by a therapist according to the individual symptoms and physical condition. Patients were requested to include the exercises in their daily routine and provide feedback about their general condition, mood, training status and gait using a visual analogue scale that ranged from 1 to 5. Via the app, therapists were able to supervise the progress and could be contacted directly. They also called the patients every 2–3 weeks for assessments and adjustments to their training programs.

### Statistical analysis

All statistical analyses were performed using SPSS software package version 24 (IBM Corp. Released 2016. IBM^®^ SPSS^®^ Statistics for Windows, Version 24.0.0.2, Armonk, NY, USA: IBM Corp.).

Normality of data was tested by Kolmogorov–Smirnov–Lilliefors test and variance homogeneity by Levene test. For normally distributed data, Pearson’s correlation was tested and paired t-test was performed in order to analyze differences from T0 and T2. Due to the fact that several parameters were not normally distributed, Spearman’s correlation was calculated for those, and Wilcoxon test was applied. To compare the groups of R and NR, independent Sample T-test was performed for normally distributed data, and Mann–Whitney U test was used when the assumption of normality was not met. The same approach was applied to compare SG and CG.

A significance level of *p* < 0.05 was used in this feasibility study. Cohen’s d is presented as measure of effect size.

## Results

All 17 participants except one (due to a non-related SAE) completed the post assessment after the training program. The mean duration between T0 and T2 was 204.3 ± 7.4 days (95-CI 200.3–208.3). Results were compared with 16 age, gender and H&Y stage matched PD patients from our database. The mean time between T0 and T2 was 423.4 ± 159.4 days (95-CI 338.5–508.4) for the CG. An overview of all assessments can be found in Table 2.

**Table 2:** Overview of all assessments

	SG					CG					*p* (between groups, (n = 16)	Cohen’s d
	T0 (n = 17)	T2 (n = 16)	n = 16)Δ in %	*p*	Cohen’s d	T0 (n = 16)	T2 (n = 16)	Δ in %	*p*	Cohen’s d		
	mean ± SD	mean ± SD		(n = 16)		mean ± SD	mean ± SD					
H&Y	2.0 ± 0.6	1.9 ± 0.5	− 3	1.00	0.0	2.1 ± 0.4	2.1 ± 0.4	+ 1.9	0.763	0.07	0.895	**− **0.05
UPDRS-III	15.3 ± 9.2	15.3 ± 7.4	± 0	0.853	0.05	12.6 ± 6.6	15.7 ± 8.5	+ 24.3	0.091	0.45	0.563	**− **0.21
PDQ-39	12.9 ± 14.1	23.6 ± 14.8	+ 83.4	**0.010**	0.74	15.4 ± 9.6	24.4 ± 16	+ 58.7	0.105	0.47	0.940	0.03
PDQ-Mob	19.5 ± 20.1	20.0 ± 19.3	+ 2.6	0.775	0.07	15.7 ± 12.6	27.8 ± 24.9	+ 77.0	0.061	0.55	0.086	**− **0.70
MOCA	25.7 ± 2.9	27.1 ± 1.7	+ 5.3	0.132	**− **0.06	26.3 ± 2.3	26.2 ± 2.5	− 0.2	1.00	0.0	0.147	0.54
TUG	8.4 ± 2.6	9.1 ± 2.3	+ 8.2	0.298	0.27	8.8 ± 2.0	8.73 ± 1.7	− 0.7	0.861	**− **0.08	0.803	0.09
2MWT	172.8 ± 26.9	166.5 ± 31.0	− 3.6	0.304	**− **0.27	169.6 ± 34.1	178.4 ± 32	+ 5.2	0.345	0.42	0.141	**− **0.55
FES-I	23.5 ± 8.4	26.8 ± 15.8	+ 14.2	0.915	**− **0.17	27.1 ± 12.7	23.8 ± 9.5	− 12.3	0.161	**− **0.38	0.897	0.05
M-ADL, surface	73.7 ± 36.9	62.3 ± 41.3	− 15.5	0.274	**− **0.28	**–**	**–**	**–**	**–**	**–**	**–**	**–**

Assessments of Smartphone Group (SG) and Control Group (CG) including all the means and standard deviations of all measured data at Baseline (T0), Intermediate Examination (T1) and Post Test (T2). Data in SG were not complete for T0: FES-I (n = 15), PDQ39 (n = 14), 2MWT (n = 14), TUG (n = 14) Paired t-test was performed for normally distributed data and Wilcoxon test for not normally distributed data to obtain *p* value from T0 to T2 in CG and SG. Unpaired t-test (normally distributed data) and Mann–Whitney U test (not normally distributed data) to compare between-group differences. Bold numbers indicate significance

### Adherence and usability with the exercise program and smartphone-app

We calculated the participation rate by dividing the days of training (monitored via the use of the application) through the total amount of days each patient was included in the study. The data transfer for patient S7 malfunctioned and is therefore excluded in the following examinations of adherence. We did not find a correlation of the training frequency (TF) with other examined clinical outcomes.

The mean TF during the study was 74.5 ± 16.9% (95%-CI 65–84), ranging from 36 to 97%. One patient trained less than 4 days per week (equals 57%) on average due to health reasons and technical issues. TF was 71% during the first 2 months (T0 to T1) with 10 patients even improving their adherence over time (t(16) = − 1.392, *p* = 0.183, d = 0.3375) indicating a successful implementation of the app-based training in their daily life.

Acceptance and feasibility were evaluated, analyzing 2 questionnaires (SUS, uMARS) and the patient-rated additional benefit.

The mean SUS score for the application “PatientConcept” was 84.5 (95%-CI 74–85) and classified as “excellent” according to Bangor et al [54], clearly exceeding the average SUS score of 68 [55]. Only two patients awarded less than 68 points due to problems with the data transfer (S7) and the lack of personal modification (S4), e.g. being able to choose the order of the exercises themselves.

“PatientConcept” scored highest in functionality (Mean = 4.3 ± 0.7) and the subjective sub score (Mean = 4.3 ± 0.4) on a scale from 0 to 5. It also got good results in Information (Mean = 4.2 ± 0.5), the app-specific subscore (Mean = 4.2 ± 0.5), Quality (Mean = 4.0 ± 0.5), Engagement (Mean = 3.6 ± 0.6) and Aesthetics (Mean = 3.8 ± 0.6).

We also asked patients to rate the additional benefit of the app-based training program on a scale from 0 (no benefit) to 10 (maximum value). Our application received a mean score of 8.2.

Furthermore, we noted the advantages the patients recognized in the usage of the application “PatientConcept” as well as ideas for improvement Additional file 3: Table 3. The often named “obligation” to train daily indicates open-mindedness towards telerehabilitation and resembles the need for individualized, telemedical applications increasing the sense of empowerment and self-efficacy of PD patients.

### Clinical scores: H&Y and UPDRS-III

The mean stage of H&Y for SG and CG patients was 2 during both examinations. The clinical important difference on the UPDRS motor score is estimated to be 2.5–5 points [56–58]. The mean score on UPDRS-III at baseline was 15.3 ± 9.2 remaining stable throughout the course of the study (post test score of 15.3 ± 7.4) in the SG (*p* = 0.85). In the CG, the UPDRS-III score increased by 3.1 points (24.3%) from 12.6 ± 6.6 in the baseline examination to 15.7 ± 8.5 (*p* = 0.091).

### Functional and sensor-based scores: TUG, 2MWT and gait parameters

SG and CG did not show any significant changes (T0 to T2) or between-group differences of the gait parameters (Additional file 2: Table 2), TUG and 2MWT (Table 2).

### Patient-reported outcome measures: M-ADL and QoL

**Fig. 1:**
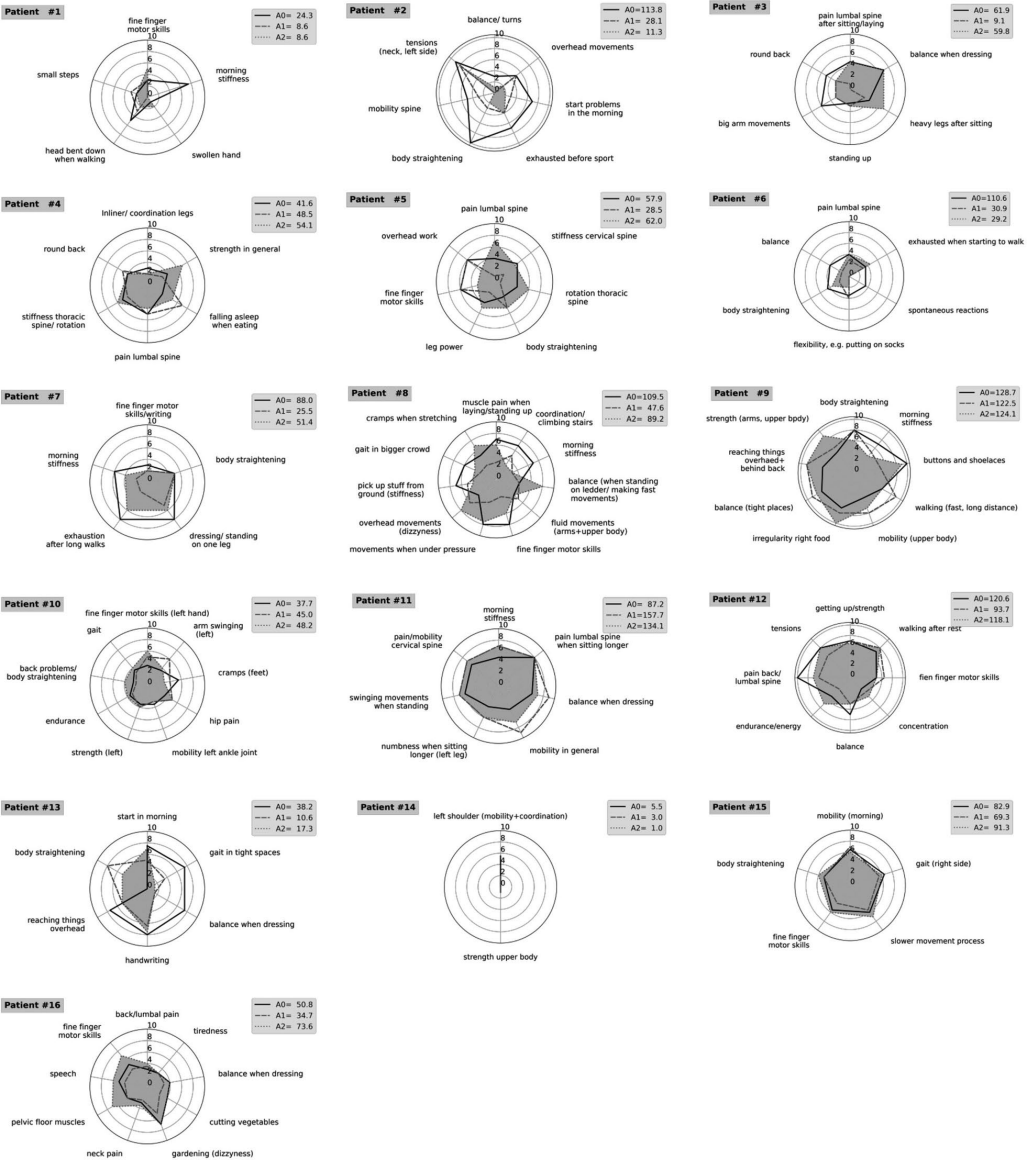
Radar plots of M-ADL scores. Radar plots of rated daily motor activity of all 16 patients. Black drawn through line represents T0, dashed line represents T1 and dotted line around grey area represents T2

The number of daily activities (M-ADL), that were affected by the motor impairment of each SG patient, ranged from 2 to 11 (Mean = 6.75) and included, e.g. body straightening, morning stiffness and fine finger motor skills. The results of the examinations (T0, T1 and T2) are represented as radar plots (Fig. 1).

The M-ADL scores significantly improved from T0 to T1 by 34.5% (*p* < 0.05) with 13 patients stating fewer motor impairments. Concerning the change of M-ADL from T0 to T2, there was an average improvement of 15.5% (*p* = 0.274).

We also used PROM’s (PDQ-39) to compare the QoL before and after the intervention but did not observe any significant changes. Both groups had a worse total score in T2: the score of the SG increased by 83.4% and the score of the CG by 58.7%. However, the results of the mobility sub score stayed almost stable for the SG (+ 2.6%) but substantially aggravated for the CG by 77% (*p* = 0.061).

### Subgroup analysis

Two subgroups were formed out of the SG according to the change of M-ADL. The R improved their scores (Mean = − 43.4%) while the NR showed an increase of impairment (Mean =  + 29%). Comparing the baseline results (Additional file 1: Table 1), we found a statistically significant difference between the R (Mean = 9.0 ± 2.9) and the NR (Mean = 6.8 ± 1.0) for the TUG, t(14) = 1.81, *p* < 0.05. The difference of both groups (R: Mean = 166.5 ± 24.6; NR: Mean = 190.0 ± 19.0) in the 2MWT was almost significant (t(14) = 2.11, *p* = 0.053). All other scores, as well as the changes from T0 to T2, did not differ significantly between the groups. Though, the group of R did show more positive changes of PDQ-Mobility, FES-I, TUG and gait velocity. The UPDRS-III score improved for the group of R by 12.1% (Mean = − 2.1 ± 1.9) while the NR showed a clinically relevant worsening of 47.6% (Mean = 5.0 ± 2.9) [56–58]. There was no significant statistical correlation between our classification in R and NR (difference of M-ADL) with any of the other scores (*p* > 0.05).

**Fig. 2:**
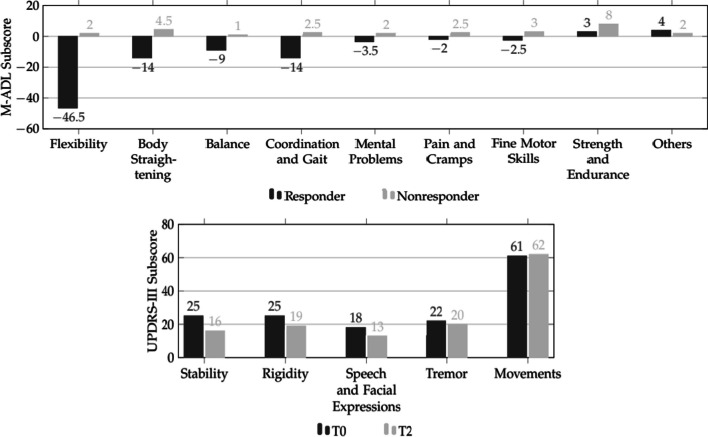
Response to training according to different subcategories. **a** M-ADL subcategories: Dark bars represent change of total score of each subcategory from T0 to T2 for R, light bars represent change for NR. **b** UPDRS-III subcategories: Dark bars represent total scores in each subcategory for all R at T0, light grey bars represent scores for all R in T2

Furthermore, we identified the type of motor symptoms that attenuated through exercise training (Fig. 2). The average improvement after the training period was highest for each ADL connected to flexibility (Mean = − 2), body straightening (Mean = − 1.9), balance (Mean = − 1.6) and coordination or gait (Mean = − 0.8).

These findings were confirmed by the results of the UPDRS-III examinations of the R where we found an improvement in stability by 36.0%, rigidity by 24.0%, speech and facial expressions by 27.8% and tremor by 9.1%, while the movement scores increased by 1.6%.

## Discussion

In this interventional feasibility study, the acceptance and initial effectiveness of a digital, home-based, high-frequency, individualized exercise program for PD patients was evaluated over a period of 6 to 8 months. To obtain an objective and comprehensive assessment, we combined a qualitative and quantitative analysis. Thus, we identified a group of training-responsive individuals (R) that profited significantly from the high-intensity training.

### Feasibility and acceptance of long-term smartphone-based exercise training

The monitored TF can be considered consequently high, detecting a slightly better adherence in the second half of the study. Although different recommendations for the training intensity in PD are described and significantly depend on the individual conditions, high-frequency training such as an average of 20–30 min of aerobic, resistance or balance training 3 to 5 days per week is broadly agreed upon [18]. Being able to implement the training in their daily routine and freely choose the time and location additionally motivates patients to train more frequently. Participants of the study also appreciated the 2-week modification of the exercises and the possibility to contact the therapist. All patients indicated the desire to continuously use the application after the study and to recommend it to other PD patients. Many of them claimed to be willing to purchase the app if subject to charge.

Due to the positive feedback, excellent scores in both SUS and uMARS, as well as the consistently high adherence, the app can be considered feasible and accepted over a longer period of time. The implementation of a permanent, smartphone-based exercise training as a supplemental treatment in PD routine care should be strongly considered and a meta-analysis combining the results of more programs as well as comparing their effectiveness should be conducted.

### Patient-reported outcome measures depict an additional benefit for a group of PD patients

When declaring an additional benefit of an intervention in the German HTA process (AMNOG), it is crucial to base it on patient-relevant endpoints including morbidity, safety, and health-related QoL [59]. As a consequence, patient-reported M-ADL served as our primary outcome monitoring the relevant effects of the intervention from the perspective of the patient. 

All patients reported an additional benefit with the smartphone-based, high-frequency training (Mean = 8.2 ± 1.3) and would recommend it to other PD patients. 10 out of 16 patients (R) showed an additional improvement of the previously individually-defined M-ADL. 

**Fig. 3:**
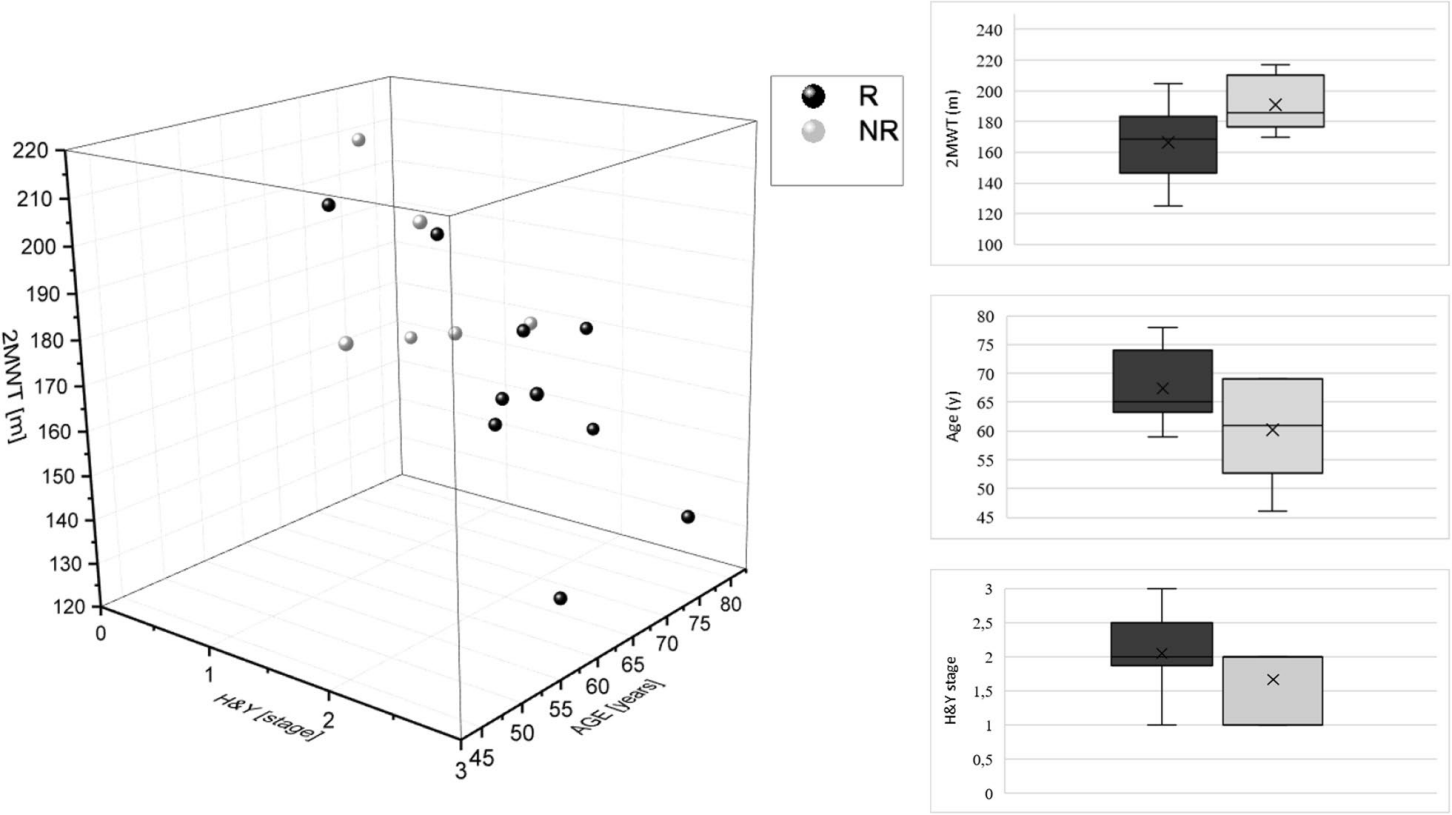
Characteristics of of R and NR. Scatter plot of R (dark) and NR (grey) according to their baseline examinations in age, stage of H&Y and maximum distance at 2MWT. Box plots present the distribution of 2MWT distance, age, and H&Y stage within the group of R and NR.

To summarize the results of the characterization (Additional file 1: Table 1), we identified that the smartphone-based exercise training lead to an additional benefit compared to standard care for older patients with severe motor symptoms (higher stage of H&Y, higher UPDRS-III, slower TUG, smaller distance at 2MWT, worse PDQ-Mobility), higher QoL (PDQ-39) and better adherence. Results from the General characteristics (Age), gait tests (2MWT), and clinical assessments (H&Y) that distinguish visibly between R and NR are presented in Fig. 3.

We furthermore identified the motor impairments (flexibility, body straightening, stability) that profited most from the training program. Previous studies and meta-analyses were able to show the modification of a wide spectrum of motor symptoms (e.g. balance, gait) through exercise therapy [15, 16, 20, 60–62]. We found recent evidence that the maximized benefit for the improvement of mobility can be achieved by targeted exercise intervention [16]. Since we focused on motor skills related to daily activities (e.g. putting on shoes) as primary outcomes, an enhacement of flexibility, body straightening and stability can be seen as a therapy success if its due to the training. Our telemedical approach, can therefore confirm the positive effects of regular exercise training on motor symptoms that has been proven in many studies [5, 9, 10, 12, 17, 60].

QoL, as a commonly used PROM and symbol for the maintainance of physical abilities, was examined for SG and CG throughout the course of the study, but did not change significantly. Corresponding with other studies [63, 64], both groups reached lower scores in PDQ-39 at T2. However, the influence of external factors such as the reinstallation of COVID19 measures, social issues or colder temperature during the post examinations (Oct.-Dec. 2021) needs to be examined in further studies [65]. Nevertheless, the results in the subsection of mobility remained stable (+ 2.6%) in the SG but worsened substantially (+ 77%) in the CG. This aligns with the other outcomes (UPDRS-III, M-ADL) related to motor skills and supports the assertion that exercise training can slow down disease progression. 

### Clinical scores for measuring gait and motor symptoms

Aiming to compile a comprehensive investigation, we objectified the patient-reported improvements by combining them with a statistical analysis of clinical scores and sensor-based gait parameters. The examination in UPDRS-III confirmed the enhancement of motor symptoms, however without being statistically significant. The distinguishing feature here was the maintenance of motor abilities by subjects in the SG, while subjects in the CG deteriorated over the 6-month follow‐up period. An average increase in UPDRS-III of 1.8 to 4.8 points annually is to be expected [58, 66]. A slight deterioration would have been expected for all study participants due to the progressive and heterogenous nature of PD. A stagnation of motor symptoms can be interpreted as a therapy success if caused by that intervention.

Complementary results of the sensor-based gait analyses did not reveal any significant changes in TUG, 2MWT or any of the measured gait parameters after the short-term (Pilot study), nor after the long-term intervention. Primarily, this indicates that training effects might not be detectable with conventional clinical scores and standardized gait analyses as they are conducted only once before and after the intervention. Due to daily variations in symptoms and reoccurring motor fluctuations, continuous or more frequent measures might be necessary to depict an accurate picture of PD patients’ current motor impairments. The execution of standardized, regular gait tests at home has shown excellent reliability and could be an option to assess gait parameters in further studies [67]. Secondly, it puts emphasis on the need to implement PROMs into clinical practice. They enable assessors to retrospectively identify the patients’ perception of the symptoms and therefore rate treatment benefits directly on the level of the recipient.

### Sustainability of discovered short-term motor improvements

Positive short-term effects of high-frequency training for PD patients are well researched and generally acknowledged [20]. The sustainability however of these effects and the benefit of long-term exercise is only marginally investigated, with a need to be examined in further studies, potentially including imaging and blood biomarkers as well.

This long-term follow-up study also aimed to compare the short- and long-term impact of the home-based, high-frequency exercise program on motor symptoms. The pilot study revealed significant improvements in patient-defined M-ADL by an of average 40%, as well as lower scores in UPDRS-III (14%) after 4 weeks of training [31]. We were able to confirm the short-term benefit of the intervention by detecting a significant improvement (35%) in the patient-defined M-ADL after 8 weeks of training (T1). 10 out of 16 patients (R) profited from the training over a longer time according to their scores of patient-defined M-ADL. The responsiveness (62.5%) in our study aligns with the proportion of responders after a perturbation (56–78%) and conventional treadmill training (74–90%) [68]. The reasons and the point of time when the beneficial impact starts to decrease as well as a more precise characterization of the R needs to be investigated in further high-quality studies.

Equally, the descriptive improvement of 14% in UPDRS-III scores that was reported after four weeks in the pilot study, was not observed after the six-month home-based exercise intervention. One reason could be the natural progression of PD that has more time to proceed and consequently deteriorate the motor activities more noticeably. External factors such as additional health problems, pharmacological adjustments or the amount of daily activity, are also indisputably more likely to change over a longer period of time and therefore change the patients’ physical and mental conditions [69]. More investigation in a larger sample size is needed to examine the influence of external factors and whether they can exceed the positive effects of regular exercising.

### Limitations

Conducting an interventional feasibility study, we mainly focused on the results and acceptance of the smartphone-based intervention in the SG. Assessors were monitoring the training and therefore not blinded. The data of the matched CG was selected from regular doctor visits and limited in terms of detailed information about the individual activity level during the investigated period. Since it was very restricted to schedule additional appointments due to the COVID19 pandemic, the average time between T0 and T2 is significantly longer for the CG than for the SG. Consequently, the between-group comparison needs to be interpreted with caution. Furthermore, the small number of participants lowers the statistical relevance of the analysis and complicates the detection of true intervention effects. Future studies should therefore include blinded assessors, an increased sample size, and an active, age-, gender- and UPDRS-matched randomized control group that is observed over the same period of time. Sustainability and long-term benefits should be explored in the process and compared to alternative programs. The distinction between responder and non-responder cannot be generalized and needs to be investigated in more depth, e.g. by adding psychiatric assessments or questioning the personal motivation to engage in digital therapy concepts, in order to identify who is benefitting most from our exercise training. The influence of external factors, such as unrelated health issues, physical activity outside the study and the emotional or social conditions should also be more closely observed.

The last limitation we encountered was the discovery of some technical issues that occurred during the long-term use of the smartphone app. Before initiating another study, this needs to be optimised so that data transfer works flawlessly for all participants. We also plan including the patients’ suggestions to enhance usability and empower them to help designing a smartphone app tailored to their needs.

## Conclusion

The results of this interventional feasibility study demonstrate that an individualized, digital, home-based, and high frequency exercise program over 6 to 8 months can be considered feasible (SUS = 84.5) and highly appreciated (personal added value = 8.2/10) by PD patients.

The individualized, smartphone-based training showed beneficial effects on patient-defined motor activities of daily living, as well as a potential to slow down the disease progress by maintaining motor symptoms and achieving higher scores in UPDRS-III and PDQ-Mobility than an active control group.

Older and motorically stronger impaired patients who indicated flexibility and body straightening as their major problems especially profited from the individualized, digital exercise program (improvement of M-ADL of 43% on average among R).

In summary, the smartphone-based training successfully combined patient-defined outcome measures with treatment tailored to the patients’ individual needs. Future studies should investigate the prospects and limitations of the application to further advance the benefits it provides PD patients in line with the health system’s continuous movement towards patient-centered care models and individualized therapy concepts.

### Supplementary Information

**Additional file 1: Table S1.** Characteristics of R and NR according to baseline examinations and outcome changes from T0 to T2.

**Additional file 2: Table S2.** Overview of all gait parameters of Smartphone Group (SG) and Control Group (CG) including all measured data at Baseline (T0), Intermediate Examination (T1) and Post Test (T2).

**Additional file 3: Table S3.** Usability of App “PatientConcept”: Positive and negative aspects of the app usage and suggestions for improvement ranked according to number of times mentioned (Tm) by patients.

## Data Availability

The data sets used and analysed during this study are available from the corresponding authors on request.

## Appendix

*Sensor-based assessments* Sensor-based gait analysis included a standardized 4 × 10 m walking test, the Time Up and Go Test (TUG) and a 2 min walking test (2MWT). The gait analysis of the CG was performed with two wearable SHIMMER2 sensors (Shimmer Research Ltd., Dublin, Ireland) attached to the outer shoe sides that were connected to a data-storing tablet via Bluetooth^®^. Signals of the sensors were recorded within a (tri-axial) accelerometer range of ± 6 g (sensitivity 300 mV/g), a gyroscope range of ± 500°/s (sensitivity 2 mV/°/s), and a sampling rate of 102, 4 Hz. During the study we used the mobile GaitLab sensors including two NilsPod (Portabiles GmbH, Erlangen, Germany) inertial instep sensors [70]. The sensor consists of a 3-d gyroscope (range ± 2000°/s), a 3-d accelerometer (range ± 16 g) and includes wireless synchronization with a sampling rate of 102.4 Hz The validity of the gait analyses with the SHIMMER2 and mobile GaitLab system was confirmed internally [71]. Gait parameters were observed to be comparable and sensor signals of both systems were transferrable to the same algorithm. Sensor raw data was stored on a tablet through a Bluetooth connection and processed by a machine learning algorithm that has been proven to be technically valid [72]. Clinically relevant spatiotemporal gait parameters were calculated [49, 50]. More comprehensive information on sensor-assissted gait analysis can be obtained from previous work [46–50]

## Abbreviations

PD: Parkinson’s disease
QoL: Quality of life
PROM: Patient reported outcome measures
H&Y: Hoehn und Yahr
SG: Smartphone group
CG: Control group
T0, T1, T2: Pre, intermediate and post test
FFQ: Frequency of falls questionnaire
FES- I: Falls Efficacy Scale International
PDQ-39: Parkinson’s Disease Questionnaire
UPDRS-III: Unified Parkinson’s Disease Rating Scale—motor score
MoCA: Montreal Cognitive Assessment
M-ADL: Motor activities of daily living
TUG: Time up and go
2MWT: 2-Minute walk test
SUS: System usability score
uMARS: User version of the Mobile App Rating Scale
R: Responder
NR: Non-responder
TF: Training frequency
SD: Standard deviation
