# Supplementary material for: The effects of an individualized smartphone-based exercise program on self-defined motor tasks in Parkinson’s disease: a long-term feasibility study
Source: J Patient Rep Outcomes. 2023 Oct 30;7:106. doi: 10.1186/s41687-023-00631-6 (PMC10616049; doi:10.1186/s41687-023-00631-6)
Supplement: Supplementary file 1 — Additional file 1: Table S1. Characteristics of R and NR according to baseline examinations and outcome changes from T0 to T2. [file 41687_2023_631_MOESM1_ESM.pptx]

## Slide 1
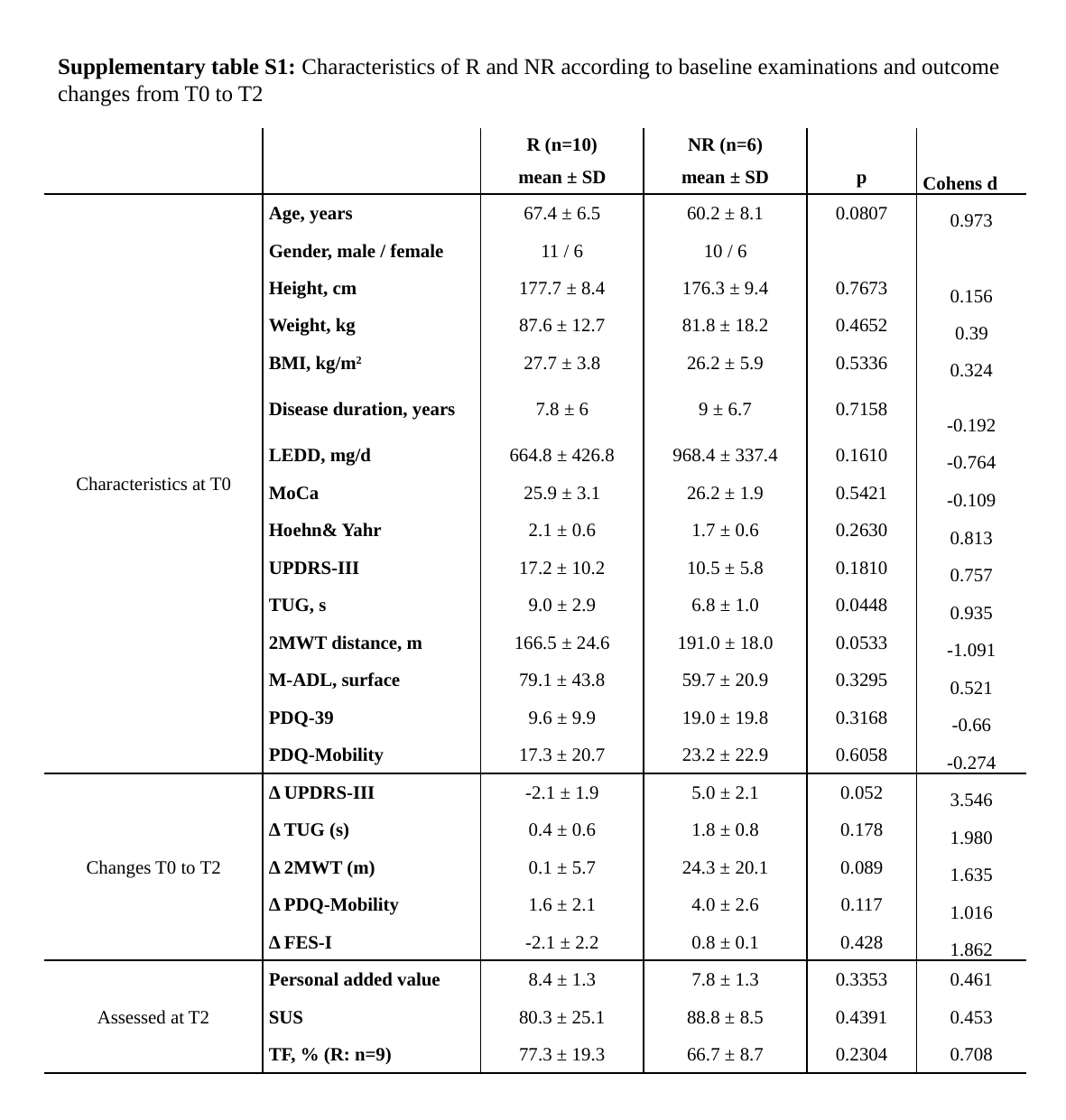

Supplementary table S1: Characteristics of R and NR according to baseline examinations and outcome changes from T0 to T2
| | | R (n=10) | NR (n=6) | | |
| --- | --- | --- | --- | --- | --- |
| | | mean ± SD | mean ± SD | p | Cohens d |
| Characteristics at T0 | Age, years | 67.4 ± 6.5 | 60.2 ± 8.1 | 0.0807 | 0.973 |
| | Gender, male / female | 11 / 6 | 10 / 6 | | |
| | Height, cm | 177.7 ± 8.4 | 176.3 ± 9.4 | 0.7673 | 0.156 |
| | Weight, kg | 87.6 ± 12.7 | 81.8 ± 18.2 | 0.4652 | 0.39 |
| | BMI, kg/m2 | 27.7 ± 3.8 | 26.2 ± 5.9 | 0.5336 | 0.324 |
| | Disease duration, years | 7.8 ± 6 | 9 ± 6.7 | 0.7158 | -0.192 |
| | LEDD, mg/d | 664.8 ± 426.8 | 968.4 ± 337.4 | 0.1610 | -0.764 |
| | MoCa | 25.9 ± 3.1 | 26.2 ± 1.9 | 0.5421 | -0.109 |
| | Hoehn& Yahr | 2.1 ± 0.6 | 1.7 ± 0.6 | 0.2630 | 0.813 |
| | UPDRS-III | 17.2 ± 10.2 | 10.5 ± 5.8 | 0.1810 | 0.757 |
| | TUG, s | 9.0 ± 2.9 | 6.8 ± 1.0 | 0.0448 | 0.935 |
| | 2MWT distance, m | 166.5 ± 24.6 | 191.0 ± 18.0 | 0.0533 | -1.091 |
| | M-ADL, surface | 79.1 ± 43.8 | 59.7 ± 20.9 | 0.3295 | 0.521 |
| | PDQ-39 | 9.6 ± 9.9 | 19.0 ± 19.8 | 0.3168 | -0.66 |
| | PDQ-Mobility | 17.3 ± 20.7 | 23.2 ± 22.9 | 0.6058 | -0.274 |
| Changes T0 to T2 | Δ UPDRS-III | -2.1 ± 1.9 | 5.0 ± 2.1 | 0.052 | 3.546 |
| | Δ TUG (s) | 0.4 ± 0.6 | 1.8 ± 0.8 | 0.178 | 1.980 |
| | Δ 2MWT (m) | 0.1 ± 5.7 | 24.3 ± 20.1 | 0.089 | 1.635 |
| | Δ PDQ-Mobility | 1.6 ± 2.1 | 4.0 ± 2.6 | 0.117 | 1.016 |
| | Δ FES-I | -2.1 ± 2.2 | 0.8 ± 0.1 | 0.428 | 1.862 |
| Assessed at T2 | Personal added value | 8.4 ± 1.3 | 7.8 ± 1.3 | 0.3353 | 0.461 |
| | SUS | 80.3 ± 25.1 | 88.8 ± 8.5 | 0.4391 | 0.453 |
| | TF, % (R: n=9) | 77.3 ± 19.3 | 66.7 ± 8.7 | 0.2304 | 0.708 |
