# Supplementary material for: The effects of an individualized smartphone-based exercise program on self-defined motor tasks in Parkinson’s disease: a long-term feasibility study
Source: J Patient Rep Outcomes. 2023 Oct 30;7:106. doi: 10.1186/s41687-023-00631-6 (PMC10616049; doi:10.1186/s41687-023-00631-6)
Supplement: Supplementary file 2 — Additional file 2: Table S2. Overview of all gait parameters of Smartphone Group (SG) and Control Group (CG) including all measured data at Baseline (T0), Intermediate Examination (T1) and Post Test (T2). [file 41687_2023_631_MOESM2_ESM.pptx]

## Slide 1
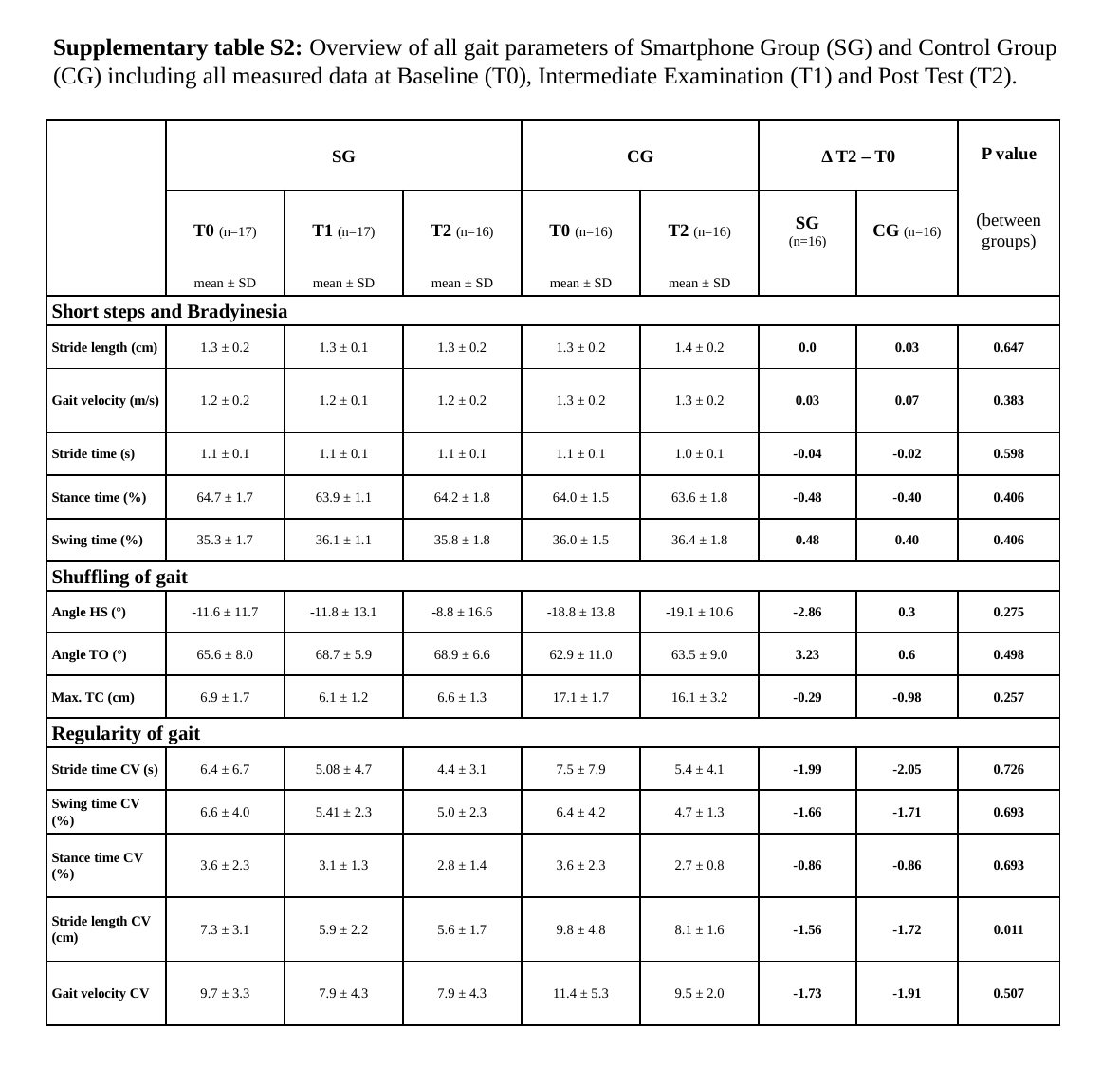

Supplementary table S2: Overview of all gait parameters of Smartphone Group (SG) and Control Group (CG) including all measured data at Baseline (T0), Intermediate Examination (T1) and Post Test (T2).
| | SG | | | CG | | Δ T2 – T0 | | P value |
| --- | --- | --- | --- | --- | --- | --- | --- | --- |
| | | | | | | | | |
| | T0 (n=17) | T1 (n=17) | T2 (n=16) | T0 (n=16) | T2 (n=16) | SG (n=16) | CG (n=16) | (between groups) |
| | mean ± SD | mean ± SD | mean ± SD | mean ± SD | mean ± SD | | | |
| Short steps and Bradyinesia | | | | | | | | |
| Stride length (cm) | 1.3 ± 0.2 | 1.3 ± 0.1 | 1.3 ± 0.2 | 1.3 ± 0.2 | 1.4 ± 0.2 | 0.0 | 0.03 | 0.647 |
| Gait velocity (m/s) | 1.2 ± 0.2 | 1.2 ± 0.1 | 1.2 ± 0.2 | 1.3 ± 0.2 | 1.3 ± 0.2 | 0.03 | 0.07 | 0.383 |
| Stride time (s) | 1.1 ± 0.1 | 1.1 ± 0.1 | 1.1 ± 0.1 | 1.1 ± 0.1 | 1.0 ± 0.1 | -0.04 | -0.02 | 0.598 |
| Stance time (%) | 64.7 ± 1.7 | 63.9 ± 1.1 | 64.2 ± 1.8 | 64.0 ± 1.5 | 63.6 ± 1.8 | -0.48 | -0.40 | 0.406 |
| Swing time (%) | 35.3 ± 1.7 | 36.1 ± 1.1 | 35.8 ± 1.8 | 36.0 ± 1.5 | 36.4 ± 1.8 | 0.48 | 0.40 | 0.406 |
| Shuffling of gait | | | | | | | | |
| Angle HS (°) | -11.6 ± 11.7 | -11.8 ± 13.1 | -8.8 ± 16.6 | -18.8 ± 13.8 | -19.1 ± 10.6 | -2.86 | 0.3 | 0.275 |
| Angle TO (°) | 65.6 ± 8.0 | 68.7 ± 5.9 | 68.9 ± 6.6 | 62.9 ± 11.0 | 63.5 ± 9.0 | 3.23 | 0.6 | 0.498 |
| Max. TC (cm) | 6.9 ± 1.7 | 6.1 ± 1.2 | 6.6 ± 1.3 | 17.1 ± 1.7 | 16.1 ± 3.2 | -0.29 | -0.98 | 0.257 |
| Regularity of gait | | | | | | | | |
| Stride time CV (s) | 6.4 ± 6.7 | 5.08 ± 4.7 | 4.4 ± 3.1 | 7.5 ± 7.9 | 5.4 ± 4.1 | -1.99 | -2.05 | 0.726 |
| Swing time CV (%) | 6.6 ± 4.0 | 5.41 ± 2.3 | 5.0 ± 2.3 | 6.4 ± 4.2 | 4.7 ± 1.3 | -1.66 | -1.71 | 0.693 |
| Stance time CV (%) | 3.6 ± 2.3 | 3.1 ± 1.3 | 2.8 ± 1.4 | 3.6 ± 2.3 | 2.7 ± 0.8 | -0.86 | -0.86 | 0.693 |
| Stride length CV (cm) | 7.3 ± 3.1 | 5.9 ± 2.2 | 5.6 ± 1.7 | 9.8 ± 4.8 | 8.1 ± 1.6 | -1.56 | -1.72 | 0.011 |
| Gait velocity CV | 9.7 ± 3.3 | 7.9 ± 4.3 | 7.9 ± 4.3 | 11.4 ± 5.3 | 9.5 ± 2.0 | -1.73 | -1.91 | 0.507 |
