# Supplementary material for: The effects of an individualized smartphone-based exercise program on self-defined motor tasks in Parkinson’s disease: a long-term feasibility study
Source: J Patient Rep Outcomes. 2023 Oct 30;7:106. doi: 10.1186/s41687-023-00631-6 (PMC10616049; doi:10.1186/s41687-023-00631-6)
Supplement: Supplementary file 3 — Additional file 3: Table S3. Usability of App “PatientConcept”: Positive and negative aspects of the app usage and suggestions for improvement ranked according to number of times mentioned (Tm) by patients. [file 41687_2023_631_MOESM3_ESM.pptx]

## Slide 1
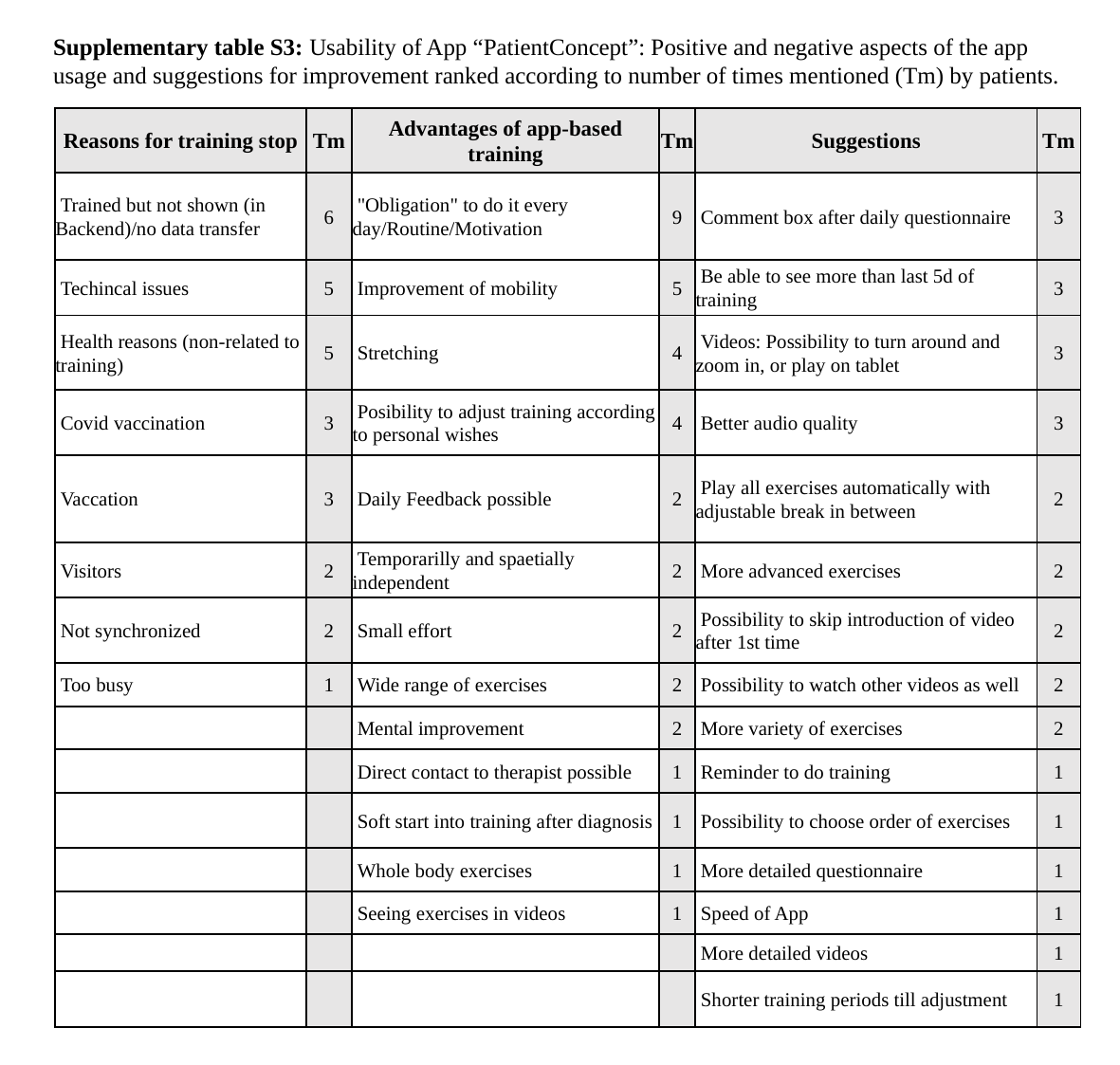

Supplementary table S3: Usability of App “PatientConcept”: Positive and negative aspects of the app usage and suggestions for improvement ranked according to number of times mentioned (Tm) by patients.
| Reasons for training stop | Tm | Advantages of app-based training | Tm | Suggestions | Tm |
| --- | --- | --- | --- | --- | --- |
| Trained but not shown (in Backend)/no data transfer | 6 | "Obligation" to do it every day/Routine/Motivation | 9 | Comment box after daily questionnaire | 3 |
| Techincal issues | 5 | Improvement of mobility | 5 | Be able to see more than last 5d of training | 3 |
| Health reasons (non-related to training) | 5 | Stretching | 4 | Videos: Possibility to turn around and zoom in, or play on tablet | 3 |
| Covid vaccination | 3 | Posibility to adjust training according to personal wishes | 4 | Better audio quality | 3 |
| Vaccation | 3 | Daily Feedback possible | 2 | Play all exercises automatically with adjustable break in between | 2 |
| Visitors | 2 | Temporarilly and spaetially independent | 2 | More advanced exercises | 2 |
| Not synchronized | 2 | Small effort | 2 | Possibility to skip introduction of video after 1st time | 2 |
| Too busy | 1 | Wide range of exercises | 2 | Possibility to watch other videos as well | 2 |
| | | Mental improvement | 2 | More variety of exercises | 2 |
| | | Direct contact to therapist possible | 1 | Reminder to do training | 1 |
| | | Soft start into training after diagnosis | 1 | Possibility to choose order of exercises | 1 |
| | | Whole body exercises | 1 | More detailed questionnaire | 1 |
| | | Seeing exercises in videos | 1 | Speed of App | 1 |
| | | | | More detailed videos | 1 |
| | | | | Shorter training periods till adjustment | 1 |
